# Skeletal Muscle Atrophy Induced by Diabetes Is Mediated by Non-Selective Channels and Prevented by Boldine

**DOI:** 10.3390/biom13040708

**Published:** 2023-04-21

**Authors:** Luis A. Cea, Walter Vásquez, Romina Hernández-Salinas, Alejandra Z. Vielma, Mario Castillo-Ruiz, Victoria Velarde, Magdiel Salgado, Juan C. Sáez

**Affiliations:** 1 Instituto de Ciencias Biomédicas, Facultad de Ciencias de la Salud, Universidad Autónoma de Chile, Av. Llano Subercaseaux 2801, San Miguel, Santiago 8910060, Chile; 2 Departamento de Fisiología, Facultad de Ciencias Biológicas, Pontificia Universidad Católica de Chile, Santiago 8330024, Chile; 3 Centro de Fisiología Celular e Integrativa, Facultad de Medicina, Universidad del Desarrollo, Santiago 7610000, Chile; 4 Escuela de Odontología, Facultad de Medicina Clínica Alemana, Universidad del Desarrollo, Santiago 7610000, Chile; 5 Escuela de Química y Farmacia, Facultad de Medicina, Universidad Andres Bello, Santiago 8370149, Chile; 6 Departamento de Ciencias Químicas y Biológicas, Facultad de Ciencias de la Salud, Universidad Bernardo O’Higgins, Santiago 8370854, Chile; 7 Instituto de Fisiología, Universidad de Valparaíso, Valparaíso 2340000, Chile; 8 Instituto de Neurociencias, Centro Interdisciplinario de Neurociencias de Valparaíso, Universidad de Valparaíso, Pasaje Harrington 287, Playa Ancha, Valparaíso 2340000, Chile

**Keywords:** sarcolemma permeability, connexins, calcium atrophy, hemichannel blocker

## Abstract

Individuals with diabetes mellitus present a skeletal muscle myopathy characterized by atrophy. However, the mechanism underlying this muscular alteration remains elusive, which makes it difficult to design a rational treatment that could avoid the negative consequences in muscles due to diabetes. In the present work, the atrophy of skeletal myofibers from streptozotocin-induced diabetic rats was prevented with boldine, suggesting that non-selective channels inhibited by this alkaloid are involved in this process, as has previously shown for other muscular pathologies. Accordingly, we found a relevant increase in sarcolemma permeability of skeletal myofibers of diabetic animals in vivo and in vitro due to de novo expression of functional connexin hemichannels (Cx HCs) containing connexins (Cxs) 39, 43, and 45. These cells also expressed P2X_7_ receptors, and their inhibition in vitro drastically reduced sarcolemma permeability, suggesting their participation in the activation of Cx HCs. Notably, sarcolemma permeability of skeletal myofibers was prevented by boldine treatment that blocks Cx43 and Cx45 HCs, and now we demonstrated that it also blocks P2X_7_ receptors. In addition, the skeletal muscle alterations described above were not observed in diabetic mice with myofibers deficient in Cx43/Cx45 expression. Moreover, murine myofibers cultured for 24 h in high glucose presented a drastic increase in sarcolemma permeability and levels of NLRP3, a molecular member of the inflammasome, a response that was also prevented by boldine, suggesting that, in addition to the systemic inflammatory response found in diabetes, high glucose can promote the expression of functional Cx HCs and activation of the inflammasome in skeletal myofibers. Therefore, Cx43 and Cx45 HCs play a critical role in myofiber degeneration, and boldine could be considered a potential therapeutic agent to treat muscular complications due to diabetes.

## 1. Introduction

Diabetes mellitus (DM) is currently one of the most prevalent chronic diseases in the world, becoming the largest epidemic of this century. Incidences of diabetes increased by ~50% in the last 10 years. The World Health Organization indicated that in 2014, around 422 million people suffered from diabetes (~8.5% of the world’s total population over 18 years-old) and estimates that this number will be doubled by 2030 [1,2]. DM is a metabolic disorder characterized by chronically elevated blood glucose levels (higher than 126 mg/dL, while the fasting condition is considered normal between 60 and 99 mg/dL) accompanied by modifications in carbohydrate, protein, and lipid metabolism. One of the relevant health issues in diabetic patients is skeletal muscle myopathy. In addition, skeletal myopathy is believed to be the main factor in the development of comorbidities, given that skeletal muscles are the main sites of glucose uptake [3] and represent ~50% of the whole-body mass. Thus, it can significantly affect glucose levels in the whole body.

Diabetic myopathy is characterized by muscular atrophy [4]. Previous studies have demonstrated that inflammatory conditions known to induce skeletal muscle atrophy, such as denervation, endotoxemia, sepsis, and chronic treatment with glucocorticoids, induce the expression of connexin hemichannels (Cx HCs). Cx HCs constitute a family of non-selective channels that allow the diffusional transfer of ions (i.e., Ca^2+^, Na^+^, and Cl^−^) and small molecules (i.e., ATP) and can permeabilize the sarcolemma of skeletal myofibers [5,6,7,8]. The latter could explain myofiber dysfunction to a great extent. Cx HCs are located at non-appositional cell membranes communicating with the intra and extracellular space, and their activity is enhanced by inflammatory conditions [9].

Diabetes presents a significant inflammatory response [10], but whether diabetes induces the expression of hemichannels (HCs) in skeletal myofibers remains unknown. We have previously described that boldine, an alkaloid extracted from an endemic Chilean tree called Boldo, blocks Cx and pannexin1 (Panx1) HCs without affecting connexin gap junctions [11,12]. This mechanism seems to explain why boldine prevents (to a great extent) renal damage and hypertension in streptozocin-induced diabetic rats [11]. Nonetheless, it remains to be demonstrated whether boldine can prevent the skeletal muscle atrophy that is observed in diabetic individuals.

Here, we found that skeletal myofibers of diabetic rats are permeabilized via Cx HCs, a process in which a P2X_7_ receptor (P2X_7_R)-dependent pathway might play a relevant role. Accordingly, the sarcolemma permeabilization observed in skeletal myofibers can be reduced by blocking Cx HCs or P2X_7_Rs. The importance of HCs was clearly demonstrated by evaluating myofibers from diabetic mice deficient in Cx43 and Cx45 expression or by blocking all these non-selective membrane channels with boldine—two conditions that prevented muscle atrophy induced by diabetes.

## 2. Materials and Methods

DMEM, Evans blue, malondialdehyde, streptozotocin, and FURA-2AM were purchased from Merck (Kenilworth, NJ, USA). Ethidium (Etd^+^) bromide was from GIBCO/BRL (Grand Island, NY, USA), fluoromount-G from Electron Microscopy Science (Hatfield, PA, USA). N-benzyl-p-toluene sulphonamide (BTS), oil red O reagent, boldine, adenosine 5′-triphosphate disodium salt hydrate (ATP), D-mannitol, and collagenase type I were obtained from Sigma (St. Louis, MO, USA). The hydrochloride form of boldine was prepared as described previously [11] and was generously provided by Härting SA (Chile). Horse serum was purchased from Thermo Scientific (Waltham, MA, USA). Anti-Cx39, -Cx43, -Cx45, -Panx1, -P2X_7_ receptor, and -NLRP3 antibodies were from Abcam (Cambridge, MA, USA). Cy2- and Cy3-conjugated goat anti-rabbit IgG were purchased from Jackson ImmunoResearch Laboratories (West Grove, PA, USA).

Two-month-old male Sprague Dawley rats (~180 g of body weight) were used. All protocols were approved by the Bioethics Committee of the Pontificia Universidad Católica de Chile (protocol N°176) in accordance with the ethical standards established in the 1964 Declaration of Helsinki and its later amendments. All efforts were made to minimize animal suffering and to reduce the number of animals used, and alternatives to in vivo techniques were implemented when was possible.

In addition, previously described muscle-specific Cx43- and Cx45-deficient mice (Cx43^fl/fl^Cx45^fl/fl^:Myo-Cre) and control mice (Cx43^fl/fl^Cx45^fl/fl^) were used. Wild-type C57 BL/6 (C57) mice of the same age were also included for comparison (Cx43^fl/fl^ Cx45^fl/fl^), and given that they were found to present no significant differences in all parameters explored in this study, we used Cx43^fl/fl^ Cx45^fl/fl^ mice as controls in order to reduce the total number of euthanized animals. All mice used in the experiments were two-month-old males, which were maintained under light:dark 12:12 cycles with food and water *ad libitum*.

Diabetes was induced in both rats and mice in a similar way. Animals were starved for 6 h and were then intraperitoneally injected with 45 mg/kg of streptozotocin (STZ). To demonstrate the development of diabetes, blood samples were taken from the tails two days after STZ injection. Animals were considered diabetic if glycaemic index values were ≥180 mg/dL. Glycaemia was measured with a portable glucometer (Accutrend sensor, Roche, Basel, Switzerland)

Four experimental animal groups were used, namely two control groups (i.e., control and control + boldine), and two diabetic groups (i.e., diabetic (STZ) and diabetic + boldine (STZ + Boldine)). Animals were kept with water and food ad libitum for 2 weeks. Then, boldine (50 mg/kg) was administered daily before midday by gavage for the last 3 weeks after STZ injection in diabetic animals.

Blood samples were collected to determine glucose levels. Blood was applied to the glycaemic indicator strip of a portable glucometer (absorbed by capillary action), and glycaemic values were recorded from the electronic readings of the equipment in mg/dL.

This procedure was carried out as previously described [5]. In brief, cross-sectional areas (CSA) of tibialis anterior (TA) muscle fibers were evaluated by fixing them in 4% (wt/vol) paraformaldehyde, staining them with hematoxylin–eosin, and analyzing them offline with ImageJ software (National Institutes of Health).

This procedure was carried out as previously described [5]. Briefly, animals were injected i.p. with Evans blue (EB^4−^, 80 mg/kg) dissolved in a sterile saline solution and kept for 5 h to stabilize. Then, animals were sacrificed, and muscles were dissected and fast-frozen in isopentane precooled in liquid nitrogen. EB^4−^ fluorescence intensities (λ excitation, 545 nm; λ emission, 595 nm) were quantified in cross-sections from intracellular regions by using a conventional Nikon Eclipse Ti fluorescent microscope.

Myofibers were isolated from flexor digitorum brevis (FDB) muscles as previously described [5]. Plantaris tendons and connective tissue were removed from anesthetized mice. FDB muscles were carefully dissected and immersed in culture medium (DMEM/F12 supplemented with 10% FBS) containing 0.2% collagenase type I, incubated for 3 h at 37 °C, and transferred to a 15 mL test tube (Falcon) with 5 mL of culture medium. Then, muscle tissue was gently triturated 10 times by using a Pasteur pipette with a wide tip to disperse single myofibers. Dissociated myofibers were centrifuged at 1000 rpm for 15 s (model 8700 centrifuge; Kubota) and washed twice by sedimentation, first in PBS solution and then in Krebs buffer (in mM: 145 NaCl, 5 KCl, 3 CaCl_2_, 1 MgCl_2_, 5.6 glucose, 10 HEPES-Na, pH 7.4), the latter containing 10 μM BTS to inhibit contractions and to reduce myofiber damage during the isolation procedure. Finally, fibers were suspended in 5 mL of Krebs HEPES buffer with 10 μM BTS, plated in plastic culture dishes or placed in 1.5 mL microcentrifuge tubes, and kept at room temperature.

To study the effect of glucose, freshly isolated myofibers were cultured in DMEN/F12 (50% each one) medium and were supplemented with glucose upon need. The final glucose concentrations were 8.0, 22.5, or 42.5 mM. Myofibers were cultured for 24 h at 37 °C, 100% humidity, 5% CO_2_, and a 95% air environment. Subsequently, skeletal myofibers were processed for immunocytochemistry or dye uptake analysis. The possible effect of hyperosmolarity in high glucose concentrations was evaluated by replacing the excess glucose with D-mannitol.

This assay was carried out as previously described [5]. In brief, freshly isolated myofibers from FDB muscles were plated onto plastic culture dishes. Myoblast cultures were washed twice with Krebs buffer solution. For time-lapse measurements of fluorescence intensity, myofibers were incubated in a recording medium containing 5 μM Etd^+^. Intensities of Etd^+^ fluorescence were recorded in regions of interest corresponding to myofiber nuclei by using a water immersion Olympus 51W1I upright microscope (Japan). Images were captured with a Retiga 13,001 fast-cooled monochromatic digital camera (12-bit; QImaging) every 30 s, and image processing was performed offline with ImageJ software (National Institutes of Health).

As previously described [13], a construct of the mouse P2X_7_R cDNA sequence cloned in pIRES-EGFP was used. The construct was transiently transfected into connexin45-knockout HeLa cells, as previously described [14] using TurboFect (Invitrogen, Waltham, MA, USA) and was used to evaluate P2X_7_R activity based on the produced intracellular Ca^2+^ signal. This assay was carried out as described [6]. Briefly, intracellular Ca^2+^ signals were analyzed in HeLa cells transfected with P2X_7_R using FURA2-AM. Cells were incubated in Krebs–Ringer buffer containing FURA2-AM (2 μM) for 50 min at 25 °C. Then, the Ca^2+^ signal was evaluated using a Nikon Eclipse Ti microscope at two excitation wavelengths (340 and 380 nm) to calculate the ratio of the recorded fluorescence emissions (340/380 ratio).

This procedure was carried out as previously described [5]. Briefly, freshly isolated myofibers from FDB muscles were fixed in 4% paraformaldehyde and were then incubated at 4 °C for 12 h with primary anti-Cx39 (1:300), anti-Cx43 (1:300), anti-Cx45 (1:300), anti-Panx1 (1:300), anti-P2X_7_R (1:250), or anti-NLRP3 (1:300) antibodies. Then, samples were rinsed four times with PBS, followed by incubation with an appropriate dilution of Cy3-conjugated goat anti-rabbit IgG antibodies. Finally, samples were rinsed with PBS, mounted with fluoromount G on glass slides, and representative images were acquired by using an Olympus fluoview 1000 confocal microscope (Tokyo, Japan).

Results were expressed as mean ± standard error (SE) and analyzed by ANOVA and a post hoc test. Results were considered significantly different when *p* < 0.05.

## 3. Results

### 3.1. Boldine Prevents the Reduction in Cross-Sectional Area of Diabetic Skeletal Myofibers and Partially Reduced the Elevated Glycaemia

**Figure 1:**
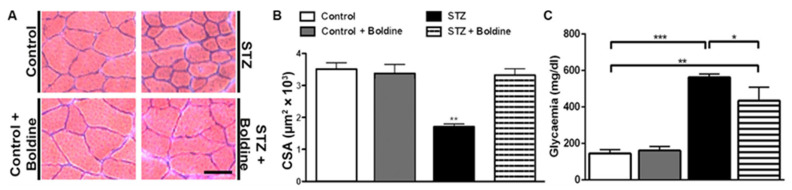
Boldine prevents the reduction in cross-sectional area of skeletal myofibers and partially prevents the elevated glycaemia in diabetic rats. (**A**) Representative hematoxylin–eosin-stained preparations of TA muscles from control and diabetic (STZ) rats treated or not with boldine. (**B**) Quantifications of images such as those shown in (**A**) (around 100 myofibers per animal were analyzed). Muscles of control (white bar), control treated with boldine (grey bar), diabetic (STZ, black bar), and diabetic treated with boldine (STZ + Boldine; 50 mg/kg, dashed bar) animals were processed for hematoxylin–eosin staining. The CSA was measured by offline analysis with ImageJ software. (**C**) Glycaemia levels were analyzed from tail blood of control and STZ rats treated or not with boldine. *** *p* < 0.001; ** *p* < 0.01; and * *p* < 0.05. Scale bar: 50 µm. (N = 5 animals per group, 10 images were taken per animal and were analyzed (around 500 fibers per group)).

We first evaluated the effect of boldine in diabetic rats. We found that glycaemia in STZ rats was at least three times higher than in control mice and that boldine treatment did not significantly affect basal glycaemia in control rats as we previously observed [11]. However, the group of diabetic rats treated with boldine had a slightly lower hyperglycaemia (not significantly different) compared to diabetic rats (in mg/dL, control: 145 ± 21; Control + Boldine: 161 ± 21; STZ: 563 ± 17; and STZ + Boldine: 452 ± 86, Figure 1C). On the other hand, glycaemia in STZ-treated rats was significantly higher compared to that of control rats (** *p* < 0.01 vs. the control. ANOVA, post hoc Bonferroni’s test).

As previously reported for other conditions, such as denervation, glucocorticoid treatment, and endotoxemia [5,6,15], skeletal myofibers from TA muscles in diabetic animals showed significant muscle reduction in the cross-sectional area (CSA) (Figure 1A, B).

Interestingly, the reduction in CSA of myofibers from diabetic rats was completely prevented by boldine treatment (Figure 1).

### 3.2. Boldine Prevents Diabetes-Induced Skeletal Myofiber Permeabilization

**Figure 2:**
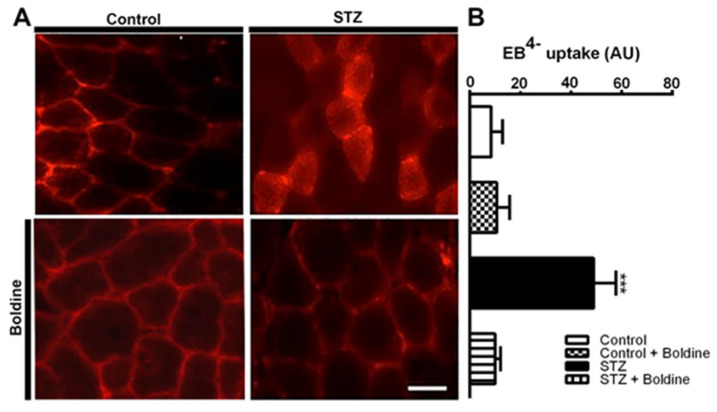
Boldine reduces the sarcolemma permeability of skeletal myofibers in diabetic rats. (**A**), Control and diabetic (STZ) rats treated or not with boldine for 3 weeks were injected i.p. with Evans blue (80 mg/kg). Tibialis anterior muscles were dissected 5 h post-injection. Evans blue (EB^4−^) was detected as red staining. Scale bar: 50 μm. (**B**), Quantification in arbitrary units (AU) of EB^4−^ inside the fibers (red signal) from images like show in (**A**), Boldine totally corrected the sarcolemma permeability. The red staining depicts the presence of the dye surrounding myofibers (control, control + boldine, and STZ + boldine) or within the myofibers (STZ). *p* < 0.001. (N = 5 animals per group and ten images per animal were analyzed (around 500 fibers per group)).

Given that the sarcolemma from skeletal myofibers in diverse inflammatory diseases that cause muscle atrophy is permeabilized via Cx HCs [5,6,15], we decided to investigate whether the sarcolemma of skeletal muscles from STZ-treated rats would also be permeabilized. To study this possibility, Evans blue was injected into control rats (EB^4−^, 80 mg/kg, i.p.) as well as diabetic rats treated or not treated with boldine. Animals were euthanized after 5 h of stabilization, after which TA muscles were isolated and fast frozen. Intense intracellular EB^4−^ staining was found in cross sections of TA muscles from diabetic rats (Figure 2, panel STZ).

### 3.3. Boldine Reduces the Distribution and Functional Expression of Non-Selective Channels in Diabetic Skeletal Myofibers

**Figure 3:**
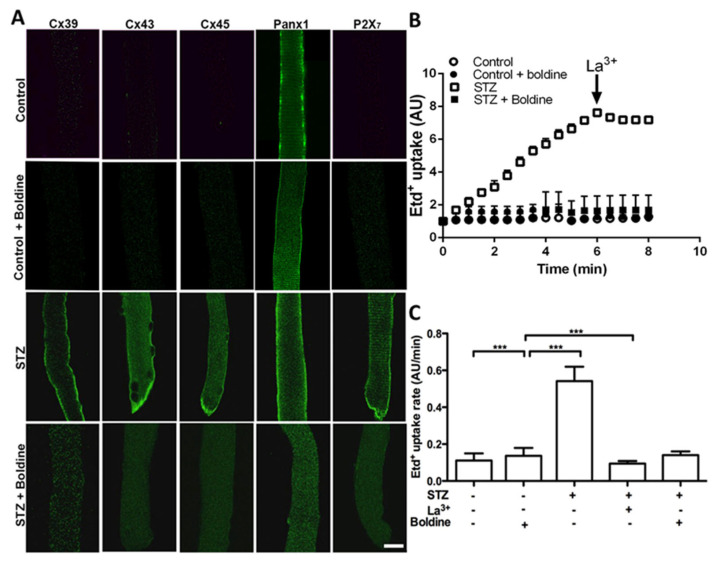
Boldine reverts the sarcolemma permeabilization of freshly isolated skeletal myofibers from diabetic rats. (**A**) Immunofluorescence against Cx39, Cx43, Cx45, P2X_7_, and Panx1 proteins was evaluated in freshly isolated skeletal myofibers from control and diabetic (STZ) rats treated or not with boldine (scale bar: 20 μm). (**B**) Time lapse of ethidium (Etd^+^) uptake assay in myofibers of flexor digitorum brevis muscles from control (circles), control + boldine (black circles), diabetic (STZ, squares) and diabetic animals treated with boldine (50 mg/kg) for 3 weeks (STZ + boldine, triangles). (**C**) Quantification of Etd^+^ uptake rate (slopes of curves like in (**A**). In some experiments, the acute effect of 150 mM La^3+^ on the uptake rate of myofibers from control and diabetic animals was evaluated. *** *p* < 0.001. (N = 5 animals per group and from each animal 10 myofibers were analyzed).

Considering that Panx1 HCs are upregulated and Cx HCs are de novo expressed with P2X_7_Rs in several muscular pathological conditions [5,15,16], we decided to study whether the sarcolemma of skeletal myofibers from diabetic animals present non-selective channels as well as changes in permeability by using indirect immunofluorescence and Etd^+^ uptake in freshly isolated skeletal muscle fibers (Figure 3).

The sarcolemma of myofibers from control rats presented Panx1 reactivity with a striation pattern but an absence of Cxs 39, 43, and 45 as well as P2X_7_R (Figure 3A). However, myofibers from diabetic (STZ-treated) rats showed diffused intracellular and intense sarcolemma reactivity for all Cxs studied as well as striated P2X_7_R reactivity. We also found that three weeks of treatment with boldine changed the distribution of Cxs (Cx39, Cx43, and Cx45) detected by immunofluorescence in isolated myofibers (Figure 3A). An evident reduction in fluorescence intensity was detected at the edge of myofibers, and most of the reactivity was detected as intracellular puncta (Figure 3A, bottom panels). Notably, reactivity for Cx39 and Panx1 showed abundant intracellular puncta in myofibers of the TA muscle from diabetic rats treated with boldine, while the Panx1 striation pattern was not evident (Figure 3A). Similar changes in Panx1 and P2X_7_R redistributions were found in myofibers of FDB muscles from diabetic rats treated with boldine (Figure 3A). In accordance with the above observations, a drastic increase in ethidium uptake was observed in myofibers from STZ-treated mice, which was significantly higher than control animals (Figure 3B,C). Importantly, this increase was entirely prevented in fibers from STZ mice treated with boldine (50 mg/kg) (dye uptake rate 0.53 ± 0.4 vs. 0.10 ± 0.05 AU/min, *p* < 0.001).

*Boldine prevents membrane permeability to Ca^2+^ by inhibiting P2X_7_ receptor*

**Figure 4:**
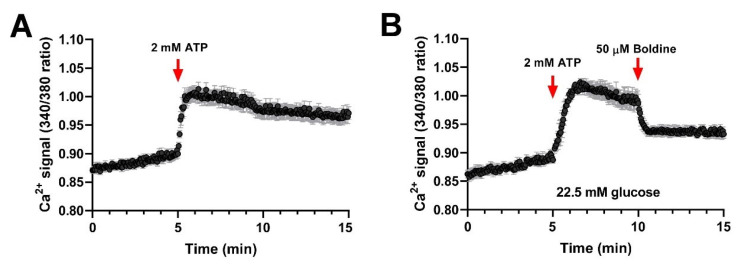
Boldine blocks P2X_7_ receptors of cells cultured in high glucose. Hela cells were transiently transfected with a P2X_7_R-EGFP vector in 22.5 mM glucose and 24 h after being loaded with Fura-2 to evaluate the P2X_7_R activity in response to boldine. (**A**) Treatment with 2 mM ATP evoked a rapid and significant increase in Ca^2+^ signal and showed a nadir for at least 10 min. (**B**). When 50 µM boldine was applied after 5 min of ATP stimulation, the Ca^2+^ signal decreased drastically. Each plotted point corresponds to the average ± SEM of the Ca^2+^ signal. In each experiment, ~20 EGFP-positive cells were recorded over time (n = 3).

Boldine is known to block Cx43, Cx45, and Panx1 HCs [11,12] but not Cx39 [7]. However, it remains unknown whether boldine affects P2X_7_Rs. We evaluated intracellular Ca^2+^ signals in HeLa cells transfected with mouse P2X_7_R by using a FURA-2 and by treating them with 2 mM ATP to activate P2X_7_Rs [17]. Notably, untransfected HeLa cells cultured in low glucose and treated with 2 mM ATP showed a very small increase in Ca^2+^ signal, but the application of a Ca^2+^ ionophore induced a clear increase in Ca^2+^ signal (Supplementary Figure S1A), consistent with the presence of a Ca^2+^ gradient across the cell membrane. In HeLa cells transfected with P2X_7_Rs, ATP elicited a significant increase in Ca^2+^ signal that was similarly blocked by 20 µM 740,003, a well-known P2X_7_R blocker [18], or by 50 µM boldine (Supplementary Figure S1A and Supplementary Figure S1B, respectively). Since diabetic animals already present elevated P2X_7_R activity, we decided to study whether boldine can block the P2X_7_R activity in Hela cell transfectants cultured in high glucose. Under these conditions, 2 mM ATP induced a clear increase in Ca^2+^ signal that slowly decreased over time (Figure 4A) and was drastically inhibited by boldine (Figure 4B).

### 3.4. The Sarcolemma of Skeletal Myofibers Is Not Permeabilized in Diabetic Mice with Myofibers Deficient in Connexin43 and Connexin45 Expression

**Figure 5:**
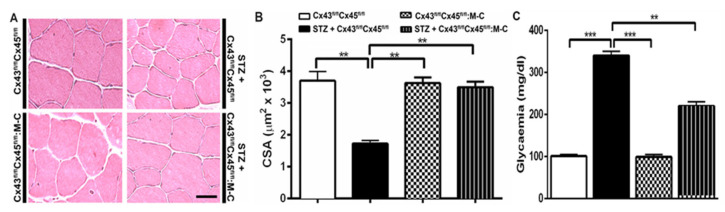
Deletion of Cx43 and Cx45 prevents skeletal muscle atrophy and partially prevents the glycaemia elevation induced by STZ in mice. (**A**) Representative hematoxylin–eosin-stained preparations of TA muscles from control (Cx43^fl/fl^Cx45^fl/fl^), control treated with STZ (STZ + Cx43^fl/fl^Cx45^fl/fl^), Cx43/Cx45 muscle-expression-deficient mice (Cx43^fl/fl^Cx45^fl/fl^:M-C), and Cx43^fl/fl^Cx45^fl/fl^: M-C + STZ mice. (**B**) Quantification of all sets of images collected as shown in (**A**). (**C**) Glycaemia levels were analyzed from tail blood of Cx43^fl/fl^Cx45^fl/fl^; STZ + Cx43^fl/fl^Cx45^fl/fl^ mice treated or not with boldine. The cross-sectional area (CSA) was evaluated to analyze the presence of atrophy. Control animals of the same age were used as controls (N = 5 animals; each value corresponds to the average ± SEM, ** *p* < 0.01 and *** *p* < 0.001. Scale bar: 50 µm.

**Figure 6:**
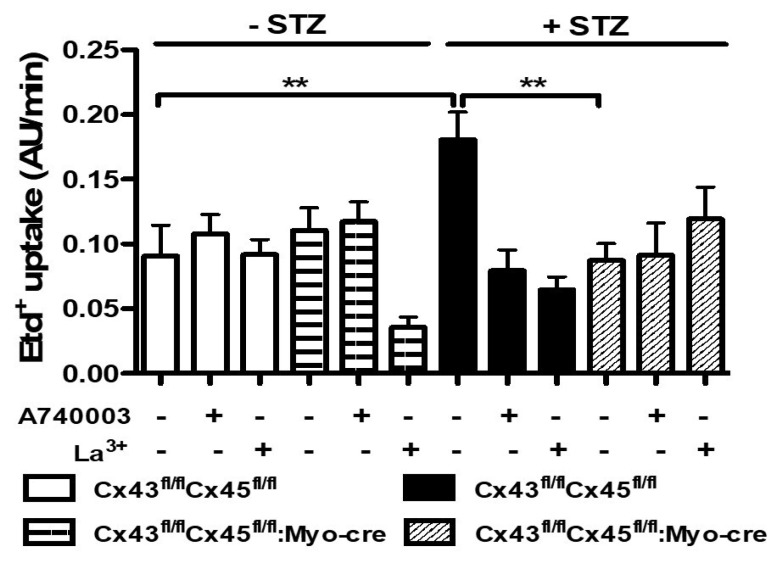
Lack of connexin43 and connexin45 expression prevents increased membrane permeability induced by diabetes in mice skeletal myofibers. Freshly isolated skeletal muscle fibers from flexor digitorum brevis muscles were isolated from control (Cx43^fl/fl^Cx45^fl/fl^) and Cx43/Cx45 muscle-expression-deficient (Cx43^fl/fl^Cx45^fl/fl^: Myo-Cre) mice, which were treated with streptozotocin (+STZ) or not (−STZ) to induce diabetes in these mice. The myofibers were used to measure membrane permeability by an ethidium (Etd^+^) uptake assay. The graph shows the Etd^+^ uptake rate obtained from slopes of fluorescent intensity over time curves. The white bar represents myofibers from control mice without STZ treatment (Cx43^fl/fl^Cx45^fl/fl^). The horizontal dashed bar represents myofibers from mice from which Cx43 and Cx45 genes were removed in early differentiation stages with the Cre-LoxP method (Cx43^fl/fl^Cx45^fl/fl^: Myo-Cre). The black bar represents myofibers from control mice (Cx43^fl/fl^Cx45^fl/fl^) treated with STZ, and the diagonal dashed bar represents myofibers from Cx43^fl/fl^Cx45^fl/fl^: Myo-Cre mice treated with STZ. Each bar represents the mean ± SE of *n* = 4 animals for each condition in independent experiments. ** *p* < 0.01.

In order to study the participation of Cx43 and Cx45 on the permeabilization of myofibers in diabetes, four groups of animals were used in this set of experiments. The glycaemia value in each group was as follows (in mg/dL): 101 ± 4 for control mice; 340 ± 10 (*p* < 0.001 with respect to all conditions) for STZ-Cx43^fl/fl^Cx45^fl/fl^ mice; 99 ± 6 for Cx43^fl/fl^Cx45^fl/fl^: Myo-Cre mice; and 220 ± 10 (*p* < 0.01 with respect to STZ-Cx43^fl/fl^Cx45^fl/fl^ mice) for STZ-Cx43^fl/fl^Cx45^fl/fl^: Myo-Cre mice. STZ treatment in control animals (Cx43^fl/fl^Cx45^fl/fl^) significantly elevated the glycaemia, and the absence of Cxs 43/45 significantly reduced the observed increase (*p* < 0.05 respect to all conditions) (Figure 5C). In these animals, we evaluated the CSA of myofibers from TA muscles. Notably, the CSA of myofibers was significantly lower only in control mice treated with STZ, whereas mice with myofibers deficient in Cx43 and Cx45 showed similar CSA to that of control mice (Figure 5) and a reduced permeabilization of myofibers (Figure 6), highlighting the role of functional Cx43 and Cx45 HCs in skeletal muscle atrophy induced by diabetes.

*High glucose-induced skeletal muscle fiber permeabilization* via *membrane channels blocked by boldine*

As shown above, diabetic animals present functional Cx HCs in muscle fibers. However, healthy muscle fibers do not present them as evaluated by immunofluorescence and do not express Cx HCs if cultured in the presence of a selective Cx HC blocker called D4 [7]. Hence, we decided to evaluate whether boldine could prevent the expression of functional Cx HCs in myofibers incubated for 24 h with 8 or 22.5 mM glucose.

**Figure 7:**
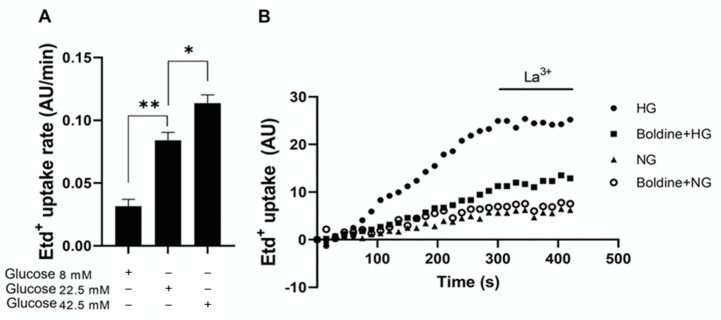
High glucose increases the sarcolemma permeability of cultured skeletal myofibers. (**A**) Etd^+^ uptake rate of myofibers cultured for 24 h in DMEM/F12 culture medium plus different glucose concentrations. Each value is the mean  ±  SEM of nuclear Etd uptake rate. * *p* <  0.05 and ** *p*  <  0.01 by ANOVA with Tukey′s multiple comparisons test. N = 3–6. (**B**) Representative fluorescence intensity curve of Etd^+^ uptake of myofibers cultured for 24 h with 8 mM glucose (NG), 8 mM glucose plus 50 µM boldine (Boldine + NG), 22.5 mM glucose (HG), or 22.5 mM glucose (HG) plus 50 µM boldine (Boldine + HG). At about 300 s recording, cells were treated with 200 µM La^3+^, a known HC blocker.

Freshly isolated myofibers showed a low Etd^+^ uptake (0.032 ± 0.09 AU/min), similar to cultures of myofibers treated with 8 mM glucose for 24 h (0.031 ± 0.05 AU/min) as well as myofibers treated with 50 µM boldine for the same period of time (0.038 ± 0.013 AU/min). However, in myofibers treated with 22.5 mM or 42.5 mM glucose (high glucose concentrations), Etd^+^ uptake was significantly higher and showed a concentration-dependent relationship in comparison to that of myofibers cultured in a medium with 8 mM glucose (Figure 7A). It can be highlighted that the fluorescence intensity of myofibers incubated in a medium containing 22.5 mM glucose was much stronger than that of control cells, and myofibers in 22.5 mM glucose treated with 50 μM boldine showed an increase in fluorescence intensity close to that of control cells (Figure 7B). The possible involvement of hyperosmolarity given by the high glucose concentration was studied using mannitol, which replaced 14.5 mM glucose. As a result, myofibers incubated for 24 h in 22.5 mM solute (8 mM glucose plus 14.5 mM mannitol) presented a dye uptake rate of 0.042 ± 0.04 AU/min, which was very close to that of the control myofibers described above.

High glucose enhances the presence of NLRP3, an inflammasome marker, which was inhibited by boldine. Given that myofibers express the molecular components of the inflammasome that become activated under an acquired pathological condition such as denervation, and because inhibition of Cx HCs prevents its activation [5], we decided to test whether high glucose could affect the inflammasome found in myofibers. To this end, we cultured myofibers as described above and tested NLRP3 immunoreactivity after 24 h of culture under control conditions treated with 22.5 mM glucose or 25.5 mM glucose plus 50 µM boldine. NLRP3 reactivity in control myofibers and in muscle fibers cultured with 22.5 mM glucose + 50 µM boldine was similar between them but lower than myofibers cultured in 22.5 mM glucose alone (Supplementary Figure S2). The latter suggests that glucose can promote the activation of the inflammasome independent of the presence of other organs and cells in a diabetic individual.

## 4. Discussion

In the present work, it was demonstrated that diabetes-induced skeletal muscle atrophy is directly associated with enhanced sarcolemma permeabilization by non-selective membrane channels, and this effect was prevented by boldine, which is an inhibitor of Cx and Panx1 HCs as well as P2X_7_ receptors.

The increase in sarcolemma permeability found in vivo using Evans blue as a permeability tracer is similar to that previously observed in denervated skeletal myofibers [5] and in skeletal myofibers of mice suffering from endotoxemia or inflammation associated with a genetic defect in dystrophin [6,15], all corresponding to in vivo demonstrations of membrane permeabilization via Cx HCs. Keeping in mind that impaired glucose transport in skeletal muscles leads to impaired whole-body glucose uptake and contributes to the pathogenesis of type 2 diabetes mellitus associated with a systemic inflammatory response [19], our findings could have a relevant impact on glucose utilization in diabetes. Related to this issue, it has been previously shown that Cx HCs play a critical role in glucose utilization in inflamed endothelial cells induced by IFN-γ plus high glucose, which augments endothelial Cx HC activity, resulting in an increase in ATP release, ATP-mediated Ca^2+^ dynamics, as well as the production of nitric oxide and superoxide anion. This also negatively alters insulin-mediated uptake, intercellular diffusion of glucose, and cell survival [20]. Consequently, it was proposed that Cx HC inhibition could prevent the activation of detrimental signaling that results in endothelial cell dysfunction and death caused by inflammatory mediators during atherosclerosis secondary to diabetes mellitus [20]. Similarly, increases in sarcolemma permeability could contribute to worsening the consequences of diabetes considering that skeletal myofibers expressing high non-selective channel activity could explain reductions in glucose utilization as well as at resting potential of skeletal muscles in diabetic individuals. This will be discussed further below.

As already mentioned, a relevant impact of diabetes on skeletal muscles is atrophy, which is common in several acquired and genetic muscular pathological conditions. To our surprise, the observed increase in Cx HC activity was also present in diabetic animals, and their inhibition or lack of expression prevented the reduction in CSA of skeletal myofibers from diabetic animals. Since Cx43 HCs and P2X_7_Rs are permeable to Ca^2+^ [21,22], it is predictable that intracellular levels of free Ca^2+^ in myofibers from diabetic animals would be high and could promote a catabolic state that may lead to atrophy, as proposed for several skeletal muscle pathologic states characterized by atrophy [5,7,15,23].

Currently, there are not many tools available to selectively block Cx HCs. Most known agents inhibit both Cx gap junction channels and Cx HCs, and the use of Cx knockout or knock-down can present limitations, given that they suppress the expression of both HCs and gap junction channels. The latter renders this technique inappropriate for determining the role of HCs in diverse physiological and pathophysiological conditions. So far, only one peptide (Gap19) is known to selectively decrease Cx43 HC activity without affecting cell coupling in cardiomyocytes subjected to metabolic inhibition [24]. However, as shown here, several non-selective channels are involved in diabetes-induced inflammation (i.e., Cx43 and Cx45 HCs and P2X_7_Rs), unveiling a limitation for the effectivity of highly selective channel blockers. In contrast, a polyvalent or super anti-inflammatory agent that simultaneously blocks several pro-inflammatory targets could be a very effective and useful resource. In this regard, boldine has been shown to block Cx and Panx1 HCs [11,12]. In the present study, we demonstrated that boldine also blocks P2X_7_Rs. It should be noted that Cx and Panx1 HCs, as well as P2X_7_Rs, have been shown to play relevant pro-inflammatory roles in diverse cell types [9,25,26], and their presence in the cell membrane should facilitate their inhibition by small organic molecules, such as boldine. Importantly, all these channels are located upstream of the intracellular inflammatory metabolic pathways, which are activated by increases in intracellular free Ca^2+^ [27]. Additionally, greater activity of these channels could establish a feed-forward mechanism in which all Panx1 and Cx HCs can mediate the release of ATP, a danger signal, and increases in Ca^2+^ influx could occur through both Cx43 HCs and P2X_7_Rs, which are Ca^2+^-permeable channels [21,22], thus activating the inflammasome. Intracellular free Ca^2+^ also increases Cx HC activity [28] through a mechanism that most likely involves the participation of Ca^2+^-dependent protein phosphorylation. This interaction might explain reductions in sarcolemma permeability induced by acutely blocking P2X_7_Rs with A740003 as observed in freshly isolated myofibers from diabetic rats.

The elevated levels of non-selective channels detected by immunofluorescence in myofibers of diabetic rats were found to be directly associated with increases in sarcolemma permeability, which most likely can be explained primarily by greater Cx43 and Cx45 HC activity. This is because boldine does not block Cx39 HCs [15], and the lack of expression of both proteins prevents the atrophic response induced by diabetes. Interestingly, boldine drastically affected the cellular distribution of all proteins studied. A similar finding was previously described to occur in mice with endotoxemia treated with boldine [6]. Although we cannot fully understand the underlying mechanism based on the available data, a possible explanation (without discarding others) could be that active channels mediate the passage of molecules or ions involved in the correct delivery of channel-forming proteins.

Cx proteins are not detected in adult normal myofibers [5,7,29], because their expression is repressed by microRNA after myoblast fusion during myogenesis [30]. However, these proteins are re-expressed by skeletal myofibers in several pathological conditions, such as denervation, Duchenne muscular dystrophy, glucocorticoid treatment, endotoxemia, and dysferlinopathy, among others [5,6,15,16,31,32]. In all these conditions, Cx HCs are the main mediators responsible for muscle atrophy [5,15,31,32] as well as muscle excitability due to a reduction in resting membrane potential [5,7,15] and inflammation of skeletal myofibers [5,16], which also occurs in diabetic muscles. This association is in line with previous studies that have reported reduced resting membrane potentials in diabetic muscles [33] and abnormal-action-potential-induced Ca^2+^ transients [34], leading to unexcitable fibers and subsequent reductions in glucose utilization due to lack of muscle contraction. Along these lines, resting membrane potential rapidly and completely recovers (30 min) in denervated muscles treated with a Cx HC blocker that prevents Cx43 and Cx45 expression in deficient myofibers followed by a drastic reduction in atrophy [7]. Similarly, we found that either inhibition of non-selective channels with boldine or preventing the expression of Cx HCs in myofibers deficient in Cx43 and Cx45 expression did not present muscle atrophy because of diabetes, suggesting that the inactivity or absence of Cx43 and Cx45 HCs would be sufficient to prevent the cascade of events leading to negative skeletal muscle outcomes. Given that greater ROS activates Cx HCs [35,36,37], the increase in sarcolemma permeability could be explained, at least in part, by the activity of de novo expressed Cx HCs in myofibers cultured with high glucose. Similarly, it has been shown that Cx HC expression in myofibers favors greater ROS production induced by glucocorticoid treatment in skeletal muscles. Additionally, the presence of ROS is significantly reduced by inhibiting Cx HCs with vitamin E [23], which was not explained by its direct redox effect. Similarly, other antioxidant agents acting as Cx HC blockers, such as resveratrol, can prevent increases in Ca^2+^ influx as well as the activation of intracellular pathways that generate free radical agents [23]. Boldine acting as a Cx HC blocker could prevent the formation of oxidant agents rather than acting as a direct antioxidant agent.

Considering that diabetes presents high levels of circulating pro-inflammatory cytokines that can induce Cx HC expression, as previously seen in endotoxemia [6], we found that high glucose itself induces permeabilization in cultured myofibers, and greater sarcolemma permeability was most likely due to the expression of Cx HCs because they are permeable to Etd^+^ and were inhibited by La^3+^ [38,39]. Interestingly, myofibers cultured in high glucose presented elevated NLRP3 levels, strongly suggesting the activation of the inflammasome as occurs in type 2 diabetes patients [40], which generates pro-inflammatory cytokines that have been shown to induce Cx expression [6]. The local inflammatory effect of high glucose on myofibers could be enhanced by circulating pro-inflammatory cytokines typically found in the systemic inflammatory condition associated with diabetes. Skeletal muscles correspond to about 50% of the body weight. Hence, the release of pro-inflammatory cytokines from myofibers could strongly contribute to the systemic inflammatory condition of diabetes as well as its numerous undesired consequences including increases in blood pressure, retinopathy, diabetic foot, and kidney dysfunction. These consequences may be prevented by inhibiting skeletal muscle Cx HCs, which would prevent the activation of the inflammasome in muscle cells. Additionally, the absence of Cx HCs in myofibers could favor glucose uptake and utilization and contribute to reducing glycaemic values. Accordingly, we found a lower glycaemic value in diabetic mice treated with boldine or with myofibers deficient in Cx43 and Cx45 expression as compared to diabetic mice that expressed these Cxs.

In summary, boldine protects skeletal muscles in diabetic animals, reducing the development of skeletal myopathies and enabling the development of normal sarcolemma functions. Thus, boldine could be used as a promising complementary treatment for diabetes.

## Data Availability

The data presented in this study are available on request from the corresponding author. The data are not publicly available due to a part of them belong to the Ph.D. thesis in development of W. Vásquez.

## Supplementary Materials

The following supporting information can be downloaded at: https://www.mdpi.com/article/10.3390/biom13040708/s1.
