# Supplementary material for: Skeletal Muscle Atrophy Induced by Diabetes Is Mediated by Non-Selective Channels and Prevented by Boldine
Source: Biomolecules. 2023 Apr 21;13(4):708. doi: 10.3390/biom13040708 (PMC10136059; doi:10.3390/biom13040708)
Supplement: Supplementary file 1 [file biomolecules-13-00708-s001.zip › biomolecules-2223247-supplementary.pdf]

Supplementary Figures

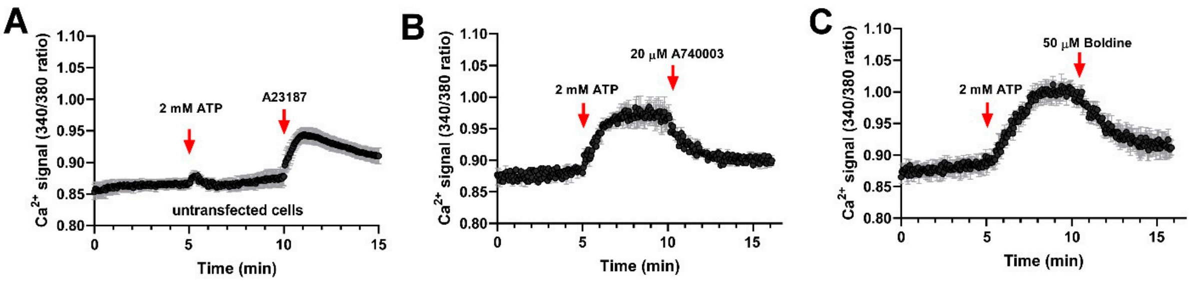

Supplementary Figure S1. Boldine blocks $P2X_7$ receptors. Hela cells transiently transfected (**B–C**) or not (**A**) with mouse $P2X_7$ receptor bound to EGFP were loaded with Fura-2 and treated with 2 mM ATP to activate the purinergic receptor. After ATP stimulation, cells were treated with 20 μM A740003 or 50 μM boldine, as denoted by the arrows. The intracellular Ca^{2+} signal evaluated as the 340/380 rate was used as an indicator of the activity of $P2X_7$Rs. The traces over time are averages ± SEM of the Ca^{2+} signal in five different experiments. In each experiment, ~20 EGFP-positive cells were recorded over time.

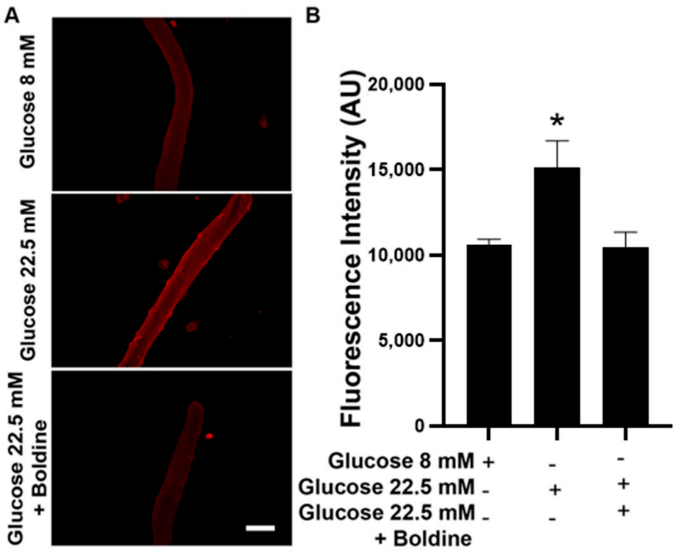

Supplementary Figure S2. Cultured skeletal myofibers treated with high glucose for 24 h present greater NLRP3 levels. Relative levels of NLRP3 (red) were evaluated by immunofluorescence using confocal microscopy. Each value is the mean ± SEM. (N = 4 mice and 12 myofibers were analyzed, for 22.5 mM 6 mice were used and 24 myofibers analyzed, for 22.5 mM 3 mice were used and 12 myofibers were analyzed, and for 22.5 M + Boldine 3 mice were used and 12 myofibers were analyzed). * $p < 0.05$ by ANOVA with Tukey´s multiple comparisons test.
